# Retraction: Association Between Mucosal Cytomegalovirus DNA Load and Short-Term Clinical Outcomes in Acute Severe Ulcerative Colitis

**DOI:** 10.7759/cureus.r258

**Published:** 2026-11-02

**Authors:** Nitesh Bassi, Dawesh Yadav, Uday C Ghoshal, Vinod K Dixit, Ujjala Ghoshal, Vinod Kumar, Anurag K Tiwari, Sayan Malakar, Aakash S Shah, Ishan Mittal, Shishirendu P Parihar, Mohita Mahajan, Maadhav Bansal, Ajay Singla

**Affiliations:** 1 Hepatology, Punjab Institute of Liver and Biliary Sciences, Mohali, IND; 2 Gastroenterology, Institute of Medical Sciences, Banaras Hindu University, Varanasi, IND; 3 Gastroenterology and Hepatology, Institute of Medical Sciences, Banaras Hindu University, Varanasi, IND; 4 Gastroenterology, Sanjay Gandhi Postgraduate Institute of Medical Sciences, Lucknow, IND; 5 Microbiology, All India Institute of Medical Sciences, Kalyani, Kalyani, IND; 6 Dermatology, Government Medical College - Amritsar, Amritsar, IND

This article has been retracted by the Editor-in-Chief. After publication, concerns were raised about the data reported in this article. A review of the study materials found that the article's multivariable analysis could not be verified from the study data, and that the unit of the primary measurement and the laboratory that performed the principal assay were reported incorrectly. The authors agree with the decision to retract.

